# Fowl adenovirus-induced different manifestations of the disease in two consecutive chicken breeding flocks in a poultry hall

**DOI:** 10.17221/27/2022-VETMED

**Published:** 2023-01-04

**Authors:** Maria Levkutova, Martin Levkut, Robert Herich, Viera Revajova, Vladimir Seman, Michaela Cechova, Mikulas Levkut

**Affiliations:** ^1^Department of Epizootology, Parasitology and Protection of One Health, University of Veterinary Medicine and Pharmacy, Kosice, Slovak Republic; ^2^Department of Morphological Disciplines, University of Veterinary Medicine and Pharmacy, Kosice, Slovak Republic; ^3^Regional Association of Veterinary Doctors, Trebisov, Slovak Republic

**Keywords:** AGE, ELISA, fowl adenovirus serotype 1, IBH, PCR, poultry

## Abstract

This study investigated an adenovirus infection in two consecutive breeding flocks in the same poultry hall. Thirty-six thousand one-day-old chickens of the ROSS 308 hybrid broiler type were kept together in one hall. The chickens in the first breeding flock during fattening did not show any clinical signs of the disease or increased mortality. Typical clinical signs of the adenovirus infection were seen in the second breeding flock. The signs included: depression, apathy, somnolence, a crouched position with a droopy head, fuzzy feathers, anaemic combs and wattles, sporadic nervous signs, and reduced weight gain. Increased mortality was recorded from 18 to 25 days of age, the higher mortality rate resulted from dehydration and exhaustion. The surviving chickens showed growth slightly below average by the end of the fattening period. The necropsies of the chickens in the first flock showed characteristic lesions for inclusion body hepatitis (IBH). Adenoviral gizzard erosions (AGE) were found mainly in the chickens of the second consecutive breeding flock. In both breeding flocks, FAdV-A was detected by polymerase chain reaction (PCR) in the liver and gizzard samples. The presence of fowl adenovirus B was not confirmed in the evaluated samples. The results showed lesions in the first flock typical for IBH, whereas the pathological changes in the second flock were characteristic of AGE.

In recent years, outbreaks of the hepatitis-hydro pericardium syndrome and adenoviral gizzard erosions have led to an increased concentration of fowl adenoviruses (FAdVs) which have become primary aetiologic agents particularly in broiler chickens (Gomis et al. 2006; Hess 2017; Revajova et al. 2017). FAdVs of the genus avian adenovirus cause inclusion body hepatitis (IBH) (Harrach et al. 2011). Twelve serotypes can be distinguished within FAdV A–E. FAdV type D or E predominantly causes IBH (Marek et al. 2010).

Some of FAdV-A strains have an unusual tissue tropism to gizzard epithelial cells with an increase in adenoviral gizzard erosion (AGE) (Schachner et al. 2018; Schachner et al. 2021). Ono et al. (2007) and Grafl et al. (2012) described both the vertical and horizontal transmission of FAdV-A, and the subsequent clinical signs and pathological changes of AGE. The new emerging FAdV-B was detected in the neighbouring Hungary and Austria, which requires further investigation (Kajan et al. 2019). Considerable economic losses caused by AGE are due to the growth retardation, higher mortality rates, and chickens with the affected gizzards being discarded in slaughterhouses (Schachner et al. 2018).

The aim of the presented case report was to check on and compare two consecutive broiler flocks in one hall for adenovirus infections (FAdV-A and FAdV-B) during 41 days of fattening.

## Case history

Adenovirus infections in broilers were monitored in two consecutive breeding flocks. Thirty-six thousand one-day-old chickens of the ROSS 308 hybrid broiler type with an average weight of 39.75 g were kept together in one hall. The stocking rate was up to 15 chickens/m^2^. The service period between two batches was 14 days and the chicken feeding hall was mechanically cleaned and chemically disinfected. The bedding consisted of straw pellets. The chickens were clinically healthy and viable. A bacteriological examination for the presence of *E.* *coli* in the intestines was performed at the establishment of the flock. The mycotic examination yielded negative results, too. The chickens’ diet did not contain animal proteins, it contained anticoccidials (Maxiban, Narazine, Nicarbazine-starter, Narazine, Nicarbazine grower I, grower II, Sacox, Salinomycin-finisher I, -finisher II without anticoccidials). The ammonia and CO_2_ concentrations were regularly measured during the fattening, which was optimal.

### VACCINATION PROTOCOL

During incubation, an *in ovo* vaccination was performed on the 18^th^ day against the infectious bursal disease virus (strain Winterfield 2512, Cevac Transmune lyophilised; Ceva-Phylaxia Co., Budapest, Hungary). In the hatchery on the 1^st ^day of life, vaccination by an aerosol against Newcastle disease (strain PHY.LMV 42, Cevac Vitapest L lyophilised; Ceva-Phylaxia Co., Budapest, Hungary) and infectious bronchitis (variant strain H120+1/96, Cevac I Bird lyophilised; Ceva-Phylaxia Co., Buda-pest, Hungary) were performed. The parents of the farmed chickens were not vaccinated against adenovirus disease or laying hen syndrome.

### CLINICAL EXAMINATION

The chickens in the first flock did not show clinical signs or increased mortality during fattening. On the other hand, typical clinical signs of adenovirus infection were seen in the second consecutive flock of broilers. The signs include depression, fuzzy feathers, apathy, somnolence, crouched position with a droopy head, anaemic combs and wattles, sporadic nervous signs, and reduced weight gain. Increased mortality was recorded from 18 to 25 days of age. The higher mortality rate resulted from dehydration and exhaustion. Slightly below-average growth was recorded in the surviving chickens by the end of fattening.

### PATHOLOGY

**Figure 1:**
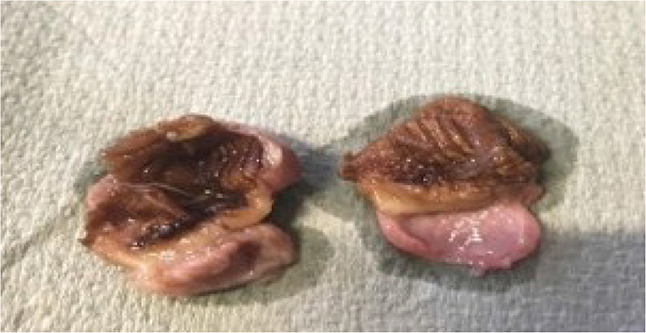
Brown erosions on the keratinoid layer of the gizzard

The pathological examination of the dead chickens showed different types of haemorrhages, from petechial to ecchymoses in the skeletal muscles, which was observed as haepatomegaly with a pale brownish-to-yellowish colour and fragile consistency. Visible miliary necrotic foci were presented throughout the tissue of the pancreas. The spleen was enlarged – splenomegaly. The small intestine exhibited catarrhal enteritis. The kidneys were oedematous with petechial haemorrhages. Gizzard lesions were found mainly in the chickens of the second flock, which were represented as multiple brown or black areas of erosions of the keratinoid layer (Figure 1) as well as inflammatory changes and ulceration underneath the gizzard mucosa.

### HISTOLOGY

**Figure 2:**
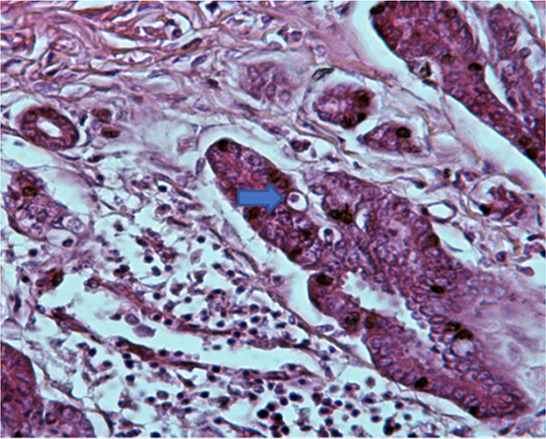
Inclusion body with a halo zone in epithelial cells (arrow) of the gizzard; H&E × 40

**Figure 3:**
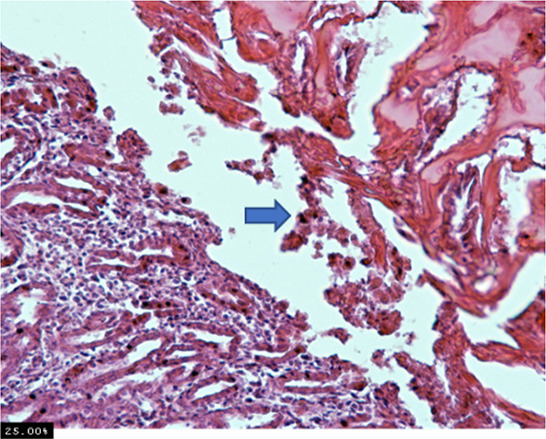
Infiltration of inflammatory cells (arrow) in the detached keratinoid layer; H&E × 40

Liver and gizzard samples were taken for a histological examination. Ten per cent neutral buffered formalin was used to fixate the samples. The fixated tissues were processed using a routine histological procedure and stained with haematoxylin and eosin. The liver samples showed intensive steatosis, with the presence of basophilic and eosinophilic intranuclear inclusions. Degeneration and necrosis of the glandular epithelial cells with inclusion bodies (Figure 2) and loss of the keratinoid layer (Figure 3) were detected in the second flock.

### SEROLOGY

An indirect enzyme-linked immunosorbent assay (ELISA) test for avian adenovirus (Fowl Adenovirus group 1 Antibody test kits; BioChek, Reeuwijk, the Netherlands) was used at days 1, 27, and 41 in the first broiler flock for serological monitoring.

The second flock was serologically evaluated at days 21 and 41.

The detection of the non-specific serotype common group antigen included 12 serotypes of avian adenoviruses.

The ELISA test confirmed seropositivity (≥ 1 071) to avian adenovirus (Table 1) in only two samples (20%) in the first flock on examination at day 41. On the other hand, the serological examination of the blood samples from ten randomly selected chickens showed a high antibody titre in all the samples in the second flock at day 41.

**Table 1:** ELISA titres of the fowl adenovirus (FAdV) in chickens from two broiler flocks

No. of chickens	Flock 1				Flock 2	
	day 1	day 27	day 41		day 21	day 41
1	1 : 4 811	1 : 277	1 : 568		1 : 690	1 : 20 973
2	1 : 8 903	1 : 7	1 : 559		1 : 1 928	1 : 19 345
3	1 : 17 720	1 : 121	1 : 935		1 : 1 249	1 : 21 322
4	1 : 21 257	1 : 7	1 : 680		1 : 910	1 : 21 322
5	1 : 12 424	1 : 21	1 : 2 047		1 : 3 448	1 : 21 322
6	1 : 20 793	1 : 24	1 : 690		1 : 4 558	1 : 21 322
7	1 : 5 785	1 : 9	1 : 735		1 : 115	1 : 21 322
8	1 : 9 241	1 : 110	1 : 2 051		1 : 325	1 : 20 985
9	1 : 18 742	1 : 97	1 : 547		1 : 338	1 : 19 526
10	1 : 14 753	1 : 8	1 : 542		1 : 1 031	1 : 21 322

### PCR TESTING

Polymerase chain reaction (PCR) testing was performed with DNA isolated from the stomach and liver tissue samples. For the DNA extraction from the tissue samples, a QIA amp DNA Mini Kit (Qiagen, Crawley, UK) was used.

The used sequences of primers were as follows – FAdV 1A: 5'-TTCGAGATCAAGAGGC CAGT-3' and FAdV 1B: 5'-GGTCGAAGTTGC GTAGGAAG-3', FAdV 5A: 5'-TACTGCCGT TTCCACATTCA-3' and FAdV 5B: 5'-AGCTGATT GCTGGTGTTGTG-3' (Niczyporuk et al. 2010). The PCR mixture contained 0.5 μM of each primer (IDT, Coralville, USA), 0.2 mM of each deoxynucleoside (Gene Craft, Ludinghausen, Germany), 2.5 mM of MgCl_2_ (Qiagen, city, UK), 1x PCR buffer (Qiagen, Crawley, UK), 2 IU Taq polymerase (Qiagen, Crawley, UK) and H_2_O to a total volume of 50 μl. The amplification was performed as follows: initial denaturation at 95 °C, 35 cycles of 94 °C for 45 s, 61 °C for 1 min, 72 °C for 2 min, and final elongation at 72 °C for 10 minutes. The samples were amplified on a Techne PTC Thermal Cycler (Techne, London, UK). The obtained products (10 μl) were separated on 1% agarose gel. The molecular mass standard (Fermentas, Vilnius, Lithuania) showed 10 bands from 1 000 bp to 100 bp.

**Figure 4:**
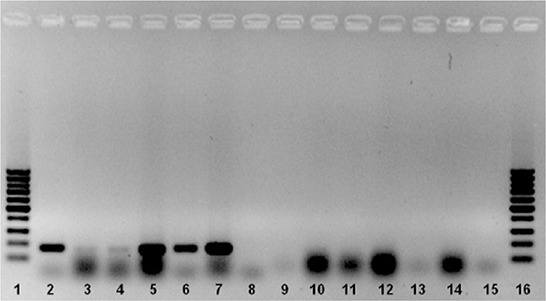
Agar gel electrophoresis of PCR products Lanes 1 and 16 DNA ladder; lanes 2–7 FAdV-A positive samples (178 bp PCR product); lane 8 and 9 negative control; lanes 10–15 FAdV-B negative samples

PCR testing revealed exclusive positivity in all the stomach and liver samples in the case of FAdV-A, confirmed by the presence of the specific PCR product of 178 bp (Figure 4). The presence of FAdV-B was not confirmed in any of the tested stomach and liver samples, and the expected 227 bp product was not visible on the agarose gel (Figure 4).

## DISCUSSION

FAdVs may be isolated from both healthy and sick chicks (Niczyporuk 2016; Adel et al. 2021). The role of adenoviruses as the primary pathogen is not clear. In our case report, broiler chickens in two consecutive flocks undergoing fattening in the same poultry feeding hall were monitored for adenovirus infections. Titres of the serological examination in chickens of the first flock demonstrated low positivity to FAdV on samplings days 21 and 41. The low serological titre was consistent with the low mortality (0.8%), without clinical manifestation induced by FAdVs. The necropsies of several dead chickens revealed lesions typical for inclusion body hepatitis. IBH is not a very serious disease with a low mortality rate ranging from 5% to 10%, although mortality can be up to 30% due to secondary infections (Mase et al. 2012). In our recent investigation of an IBH outbreak with the findings of FAdV-A (Revajova et al. 2017), the daily mortality during the culmination of the disease was only 0.8%. The second consecutive broiler flock in our current case report showed high positivity to FAdV in all the examined chickens at sampling day 41. However, it is not clear why the chickens in the second flock showed higher FAdV titres and manifested gizzard lesions. The morbidity and mortality were higher than in the first flock and reached 6% during the culmination of the disease. The necropsies revealed gizzard lesions represented as multiple brown or black areas of erosions of the keratinoid layer as well as inflammatory changes and ulceration underneath the gizzard mucosa.

The PCR analysis documented that both broiler flocks were infected with FAdV-A. Vertical and horizontal modes of FAdV-A transmission have both been described (Grafl et al. 2012; Revajova et al. 2017; Schachner et al. 2018). Based on the serological evaluation of our data, we suggest the horizontal transmission of FAdV-A in both feeding flocks.

Similarly, our results suggest that the pathogenicity of the same strain in both flocks was different. The pathogenicity of adenoviruses may vary among strains belonging to the same serotype (Absalon et al. 2017). However, our PCR analysis revealed that both outbreaks were initiated by fowl adenovirus serotype 1. The interval time between the finishing and starting the consecutive flocks of broiler feeding was 14 days. This period is necessary for preparing (cleaning and disinfecting) the feeding hall for another chicken flock. The mortality and severity of adenoviral infections are affected by factors, such as the chicken breed, the status of the immune system, or concurrent infections with other immunosuppressive infectious agents (Toro et al. 2006; Wani et al. 2014).

In conclusion, our serological data, mortality analysis, and histological evaluation of two consecutive broiler fattening flocks revealed varying serological titres, changes in the mortality rate, and a different pathological manifestation. The first flock with low serological titres demonstrated lesions characteristic of IBH. On the other hand, the second flock with high serological titres showed lesions typical for AGE. Both flocks were infected with fowl adenovirus serotype 1.

## References

[R1] Absalon AE, Morales-Garzon A, Vera-Hernandez PF, Cortes-Espinosa DV, Uribe-Ochoa SM, Garcia LJ, Lucio-Decanini E. Complete genome sequence of a non-pathogenic strain of fowl adenovirus serotype 11: Minimal genomic differences between pathogenic and non-pathogenic viruses. Virology. 2017 Jan 15;501:63-9. 27865971 10.1016/j.virol.2016.11.006

[R2] Adel A, Mohamed AAE, Samir M, Hagag NM, Erfan A, Said M, Arafa AES, Hassan WMM, El Zowalaty ME, Shahien MA. Epidemiological and molecular analysis of circulating fowl adenoviruses and emerging of serotypes 1, 3, and 8b in Egypt. Heliyon. 2021 Nov 15;7(12):e08366. 34977398 10.1016/j.heliyon.2021.e08366 PMC8683735

[R3] Gomis S, Goodhope AR, Ojkic AD, Willson P. Inclusion body hepatitis as a primary disease in broilers in Saskatchewan, Canada. Avian Dis. 2006 Dec;50(4):550-5. 17274293 10.1637/7577-040106R.1

[R4] Grafl B, Aigner F, Liebhart D, Marek A, Prokofieva I, Bachmeier J, Hess M. Vertical transmission and clinical signs in broiler breeders and broilers experiencing adenoviral gizzard erosion. Avian Pathol. 2012 Dec;41(6):599-604. 23237373 10.1080/03079457.2012.740614

[R5] Harrach B, Benko M, Both GW, Brown M, Davison AJ, Echavarria M, Hess M, Kajon A, Lehmkuhl HD, Mautner V, Mittal SK, Wadell G. Virus taxonomy: Classification and nomenclature of viruses. Ninth report of the International Committee on Taxonomy of Viruses. San Diego: Elsevier; 2011. p. 95-111.

[R6] Hess M. Commensal or pathogen – A challenge to fulfil Koch’s postulates. Br Poult Sc. 2017 Feb;58(1):1-12. 27724044 10.1080/00071668.2016.1245849 PMC5359748

[R7] Kajan GL, Affranio I, Tothne Bistyak A, Kecskemeti S, Benko M. An emerging new fowl adenovirus genotype. Heliyon. 2019 May 25;5(5):e01732. 31193583 10.1016/j.heliyon.2019.e01732 PMC6536733

[R8] Marek A, Gunes A, Schulz E, Hess M. Classification of fowl adenoviruses by use of phylogenetic analysis and high-resolution melting-curve analysis of the hexon L1 gene region. J Virol Methods. 2010 Dec;170(1-2):147-54. 20869988 10.1016/j.jviromet.2010.09.019

[R9] Mase M, Nakamura K, Minami F. Fowl adenoviruses isolated from chickens with inclusion body hepatitis in Japan, 2009–2010. J Vet Med Sci. 2012 Aug;74(8):1087-9. 22516693 10.1292/jvms.11-0443

[R10] Niczyporuk JS, Samorek-Salamonowicz E, Czekaj H. Incidence and detection of aviadenoviruses of serotypes 1 and 5 in poultry by PCR and duplex PCR. Bull Vet Inst Pulawy. 2010 Nov;54:451-5.

[R11] Niczyporuk JS. Phylogenetic and geographic analysis of fowl adenovirus field strains isolated from poultry in Poland. Arch Virol. 2016 Jan;161(1):33-42. 26446890 10.1007/s00705-015-2635-4

[R12] Ono M, Okuda Y, Shibata I, Sato S, Okada K. Reproduction of adenoviral gizzard erosion by the horizontal transmission of fowl adenovirus serotype 1. J Vet Med Sci. 2007 Oct;69(10):1005-8. 17984586 10.1292/jvms.69.1005

[R13] Revajova V, Herich R, Seman V, Levkut M Jr, Levkutova M, Karaffova V, Levkut M. An unusual outbreak of inclusion body hepatitis on a broiler chicken farm: A case report. Vet Med-Czech. 2017 Sep;62(11):631-5.

[R14] Schachner A, Matos M, Grafl B, Hess M. Fowl adenovirus-induced diseases and strategies for their control – A review on the current global situation. Avian Pathol. 2018 Apr;47(2):111-26. 28950714 10.1080/03079457.2017.1385724

[R15] Schachner A, Grafl B, Hess M. Spotlight on avian pathology: Fowl adenovirus (FAdV) in chickens and beyond – An unresolved host-pathogen interplay. Avian Pathol. 2021 Feb;50(1):2-5. 32795192 10.1080/03079457.2020.1810629

[R16] Toro H, Ewald S, Hoerr FJ. Serological evidence of chicken infectious anemia virus in the United States at least since 1959. Avian Dis. 2006 Mar;50(1):124-6. 16617995 10.1637/7442-092205R.1

[R17] Wani MY, Dhama K, Shyma K, Latheef SK, Sing SD, Tivari R. Correlation between cytokine profile, antibody titre and viral load during subclinical chicken anaemia virus infection. Vet Med-Czech. 2014 Jan;59(1):33-43.

